# ATP-Binding Cassette Systems of *Brucella*

**DOI:** 10.1155/2009/354649

**Published:** 2010-02-11

**Authors:** Dominic C. Jenner, Elie Dassa, Adrian M. Whatmore, Helen S. Atkins

**Affiliations:** ^1^Department of Biomedical Sciences, Defence Science and Technology Laboratory, Porton Down, Salisbury, Wiltshire SP4 0JQ, UK; ^2^Départment de Microbiologie, Institut Pasteur, 25 rue Dr Roux, 75724 Paris Cedex 15, France; ^3^FAO/WHO Collaborating Centre for Brucellosis, OIE Brucellosis Reference Centre, Veterinary Laboratories Agency, Addlestone, Surrey KT15 3NB, UK

## Abstract

Brucellosis is a prevalent zoonotic disease and is endemic in the Middle East, South America, and other areas of the world. In this study, complete inventories of putative functional ABC systems of five *Brucella* species have been compiled and compared. ABC systems of *Brucella melitensis* 16M, *Brucella abortus* 9-941, *Brucella canis* RM6/66, *Brucella suis* 1330, and *Brucella ovis* 63/290 were identified and aligned. High numbers of ABC systems, particularly nutrient importers, were found in all *Brucella* species. However, differences in the total numbers of ABC systems were identified (*B. melitensis*, 79; *B. suis*, 72; *B. abortus* 64; *B. canis*, 74; *B. ovis*, 59) as well as specific differences in the functional ABC systems of the *Brucella* species. Since *B. ovis* is not known to cause human brucellosis, functional ABC systems absent in the *B. ovis* genome may represent virulence factors in human brucellosis.

## 1. Introduction

*Brucella* species are the causative agents of brucellosis, the world's most prevalent zoonotic disease, with high occurrences in endemic areas including the Middle East, Asia, Mexico, and the Mediterranean [1]. The bacteria are small nonmotile, Gram-negative, nonspore-forming coccobacilli that reside within the subphylum *α*-proteobacteria, which also includes nitrogen-fixing bacteria of the genus *Nitrobacter, Rhizobium, Agrobacterium*, and *Rickettsia *[2]. They are considered facultative intracellular pathogens. 

There are six traditionally recognised *Brucella *species that have different host preferences: *Brucella melitensis *(which usually infects sheep and goats), *Brucella abortus* (cattle), *Brucella suis *(pigs), *Brucella ovis* (sheep), *Brucella canis *(dogs), and *Brucella neotomae *(desert wood rats). Furthermore, there are three newly identified *Brucella *species isolated from marine mammals: *Brucella pinnipedialis* (seals) [3],* Brucella ceti *(dolphins and porpoises) [3], and *Brucella microti* (voles) [4]. Although *Brucella *are primarily animal pathogens causing infectious abortions in females and orchitis in males [5], four of the nine species may infect humans (*B. melitensis*, *B. abortus, B. suis,* and occasionally *B. canis*, in order of disease severity) causing a range of flu-like symptoms including fever, sweats, malaise, and nausea [6]. Transmission to humans takes place via three recognised channels: (i) the consumption of infected animal products, (ii) direct contact with infected animal birth products, and (iii) the inhalation of aerosolised *Brucella*. Due to the nature of the human disease and the ability to be infectious via aerosol, *Brucella* species have been classified as category B threat agents by the US Centre for Disease Control and Prevention (CDC) [7]. 

Genome sequence analysis of *B. melitensis *16M [8], *B. suis* 1330 [9], *B. abortus* 9-941 [10], *B. canis *RM6/66 (NCBI: NC_009504 and NC_009505, unpublished), and *B. ovis* 63/290 (NCBI: NC_010103 and NC_0010104, unpublished) has demonstrated the close relatedness of these organisms [11, 12]. The genomic DNA of each strain comprises two chromosomes of approximately 2.1 Mb and 1.2 Mb. DNA-DNA hybridisations between the species had previously revealed over 90% similarity between the species, leading to the suggestion that all *Brucella *species should be classified as *B. melitensis *[13, 14]. However, it is widely believed that the differences in host specificity and pathogenicity are related to *Brucella* genetics; although there is currently little experimental evidence to support this, a few studies have found differences between the *Brucella* species genomes that may support this hypothesis [10, 15, 16]. A significant proportion of the *Brucella *genomes appear to code for ATP-binding cassette (ABC) systems.

ABC transporters are responsible for the import and export of many different substances across cellular membranes [17]. Although ABC transporters are extremely versatile, they all contain one defining feature, the ability to hydrolyse ATP to ADP, providing the energy needed for active transport. ABCs have three main conserved motifs known as Walker A (G-X-X-G-X-G-K-S/T, where X represents any amino acid residue), Walker B (ø-ø-ø-ø-D, where ø designates a hydrophobic residue), and a signature sequence (LSGGQ) [18]. The Walker A and Walker B motifs form tertiary structure enabling ATP-binding and can be found in all ATP-binding molecules. The signature sequence is well conserved in all ABC proteins and is also known as the linker peptide or C motif [19]. Although the configuration of ABC systems varies, the majority of ABC systems comprise of two hydrophilic ABC domains associated with two hydrophobic membrane-spanning domains (inner membrane (IM) proteins). Import systems are only found in prokaryotic organisms and contain both ABC domains and IM domains, along with extra-cytoplasmic binding proteins (BPs) designed to bind the specific allocrite of that ABC system. In Gram-negative bacteria the BPs are located in the periplasm whereas, in Gram-positive bacteria, they are anchored to the outer membrane of the cell via N-terminal lipid groups [20]. ABC systems import a diverse range of substrates into the bacterial cell including peptides [21], polyamines [22], metal ions [23], amino acids [24], iron [25], and sulphates [26]. In comparison, ABC systems involved in export functions usually contain only IM and ABC domains fused together via either the N-terminus (IM-ABC) or the C-terminus (ABC-IM), which homodimerise to create a functional system [27]. Substances exported by ABC transporters include antibiotics in both producing and resistant bacteria [28, 29], fatty acids in Gram-negative bacteria [27], and toxins [30]. In addition to transporters, many ABC proteins have roles in house-keeping functions, such as regulation of gene expression [31] and DNA repair [27, 32]. These proteins do not contain IM domains but are constituted of two fused ABC domains (ABC2) [27]. There is now increasing evidence that ABC systems can play roles in bacterial virulence [33–36] and can be used as targets for vaccine development [37]. 

The recent sequencing of the genomes of *B. melitensis *16M [8], *B. abortus* 9-941 [10], *B. suis* 1330 [9], *B. ovis* 63/290 (NCBI: NC_009504 and NC_009505, unpublished), and *B. canis* RM6/66 (NCBI: NC_010103 and NC_0010104, unpublished) has enabled the genomic comparison of different *Brucella* species. We report the creation and comparison of reannotated inventories of the functional ABC systems in *Brucella*. This improved annotation has assisted in understanding *Brucella* lifestyles and the identification of ABC systems that may be involved in virulence.

## 2. Methods

The prediction of ABC systems in sequenced bacterial genomes is based on annotation- and similarity-based homology assessment of identified or predicted ABC proteins from heterologous bacterial systems. The Artemis viewer (available from http://www.sanger.ac.uk) was used to visualise the sequenced genomes of *B. melitensis *16 M, *B. suis *1330, *B. abortus *9-941, *B. canis *RM6/66, and *B. ovis *63/290 [8–10]. Using the annotated genomes, ABC proteins were searched for using an array of related words, specifically “ATP-binding cassettes,” “binding protein”, or “outer membrane protein.” For completeness all proteins that were labelled as hypothetical or conserved hypothetical proteins were also checked. Hits from this search were compiled and then genes upstream and downstream were also checked to ensure that all genes from one system were found. After the genome searches were completed, protein sequences were aligned using the basic local alignment search tool (BlastP) against other ABC proteins using the ABC systems: Information on Sequence Structure and Evolution (ABCISSE) database [27, 38]. The ABCISSE database comprises 24000 proteins from 9500 annotated systems over 795 different organisms. Proteins searched against ABCISSE that scored a threshold *e*-value of 10^−6^ were assigned to an ABC family and subfamily based on the hits from the ABCISSE database. Where searches on ABCISSE were unclear or hits for multiple families were produced, proteins were aligned using BlastP searches against the Genbank protein database. Use of this larger database increased the number of positive hits and functions that could be assigned. An ABC system was defined as a series of contiguous ORFs that shared the same family, subfamily, and substrate. A complete signal sequence (LSGGQ) was identified in the majority of the ABC proteins identified, and all of the other ABC proteins contained remnants of a complete signal sequence. Walker A and Walker B sequences were not sought during these searches. 

The ABC system inventories compiled in this study include systems that contain genes with predicted frame shift mutations and premature stop codons. For example, the *B. melitensis* 16M gene BMEII0099 is a known pseudogene with multiple premature stop codons. However, this gene is part of an ABC system that is encoded by another four genes (BMEII0098, BMEII00101, BMEII102, and BMEII0103), all of which are predicted to be functional; the mutation in BMEII0099 might render the whole system nonfunctional or it is possible that the other four genes could create a partially functional system. Due to the inability to determine the functionality of ABC systems using bioinformatic techniques, the ABC systems where one or more components were predicted to be nonfunctional were excluded from the total ABC system numbers and functions of the ABC systems. Within the genomes of all *Brucella* species single components of ABC systems (mainly BP) not attached to individual systems were located. These were included in ABC system inventories and termed lone components but were not included in total functional ABC system counts.

## 3. Results and Discussion

The genome structures of *Brucella *species are very similar [10–12], and although it is widely believed that the differences in *Brucella* species virulence and host preferences are related to their genetic composition, there is little experimental evidence to support this belief. However, there are a few studies that have uncovered differences between the genomes [10, 15, 16]. In this study we have compared the presence of putative functional ABC systems in the genomes of *B. melitensis *16M (BM), *B. suis *1330 (BS), *B. abortus* 9-941(BS), *B. canis* RM6/66 (BC), and *B. ovis *63/290 (BO). In the original annotations of these genomes, a uniform nomenclature was not used and functional assignment of the systems varied considerably. Here we describe a reannotation of the ABC systems of these bacterial strains, leading to new predicted functions of the systems and predictions about how the individual components combine to form functional systems. Complete inventories of the ABC systems of BM, BS, BA, BC, and BO are shown in Table 1.

The *Brucella *strains investigated in this study all have approximately 3.3 Mb genomes comprising two chromosomes of approximately 2.1 Mb and 1.2 Mb. The total number of predicted functional ABC systems encoded by the genomes of the *Brucella* strains is similar but does show some variability (BM = 79, BS = 72, BA = 64, BC = 74, BO = 59). Our evaluation of the *Brucella* genomes confirms that these species encode a relatively high proportion of ABC system genes when compared to other bacteria [39], with an average of 8.8% of their genomes dedicated to predicted functional ABC system genes (if lone components and mutated genes are included this figure increases to 9.3%). This may reflect their relatedness to environmental *α*-proteobacteria such as *Nitrobacter *and *Agrobacterium* which also encode high numbers of ABC systems [39] that may assist in their survival in diverse conditions.

This work reports the first full inventories of ABC systems within five genome-sequenced *Brucella* strains. There are a number of specific ABC systems/genes that have previously been identified in the published literature. For example, Paulsen et al. describe two ABC systems that are present in *B. suis* and absent in *B. melitensis*. The first of these is an ABC importer encoded by BR0952 (IM), BR0953 (IM), and BR0955 (BP) [9]. Although this particular system is listed in the inventory, the ABC protein component of the system was not located in the BS genome and so this system was deemed incomplete and unlikely to be functional. The system was almost completely missing from the BM genome which is consistent with the findings of Paulsen et al. [9]. The second reported system is encoded by BRA0630, BRA0631, BRA0632, BRA0633, BRA0634, and BRA0635. However, when these genes were assessed using ABCISSE, only two of the five genes were predicted to be ABC transporter binding proteins (BRA0631 and BRA0632) and no other ABC components were located. Thus we deem this system also likely to be nonfunctional. Other genes that have been identified in the literature are BRA1080 (a dipeptide ABC transporter protein indentified in BS), BMEI1742 (a mitochondrial export ABC transporter identified in BM), and BRA0749-BRA0750 (involved in oligopeptide import) [10], all of which are present in our inventories.

## 4. ABC System Functions

In this study, we have classified the ABC systems of BM, BS, BA, BC, and BO into classes, families, and subfamilies according to the functional classification system described by Dassa and Bouige [27] (Table 2). The *Brucella *strains encode 8–12 class 1 systems, characterised by an ABC-IM domain fusion and comprising predicted export systems, and 5 class 2 systems, characterised by a duplicated fused ABC and with predicted functions in antibiotic resistance and house-keeping functions. However, we have found that most of the ABC systems of *Brucella* species belong to class 3 with roles predicted in import processes. The further classification of *Brucella *ABC systems into families and subfamilies shows that there are a high number of ABC systems of specific importer families, particularly the MOI (minerals and organic ions), MOS (monosaccharide), OPN (oligopeptides and nickel), OSP (oligosaccharides and polyols), and OTCN (osmoprotectants taurine cyanate and nitrate) families, all of which primarily function to acquire nutrients. 

**Figure 1:**
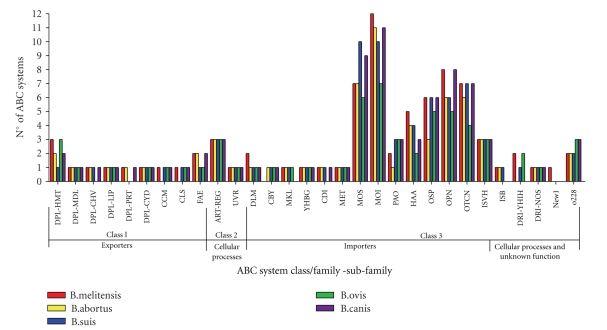
ABC system class/family-subfamily.

The predicted functionality of the ABC systems within the *Brucella* genomes is dominated by ABC systems involved in the import of nutrients (Figure 1), and although this is not uncommon amongst bacteria, it is probable that *Brucella* species utilise ABC transporters to provide most of the nutrients they require [8, 39]. In support of the findings of Paulsen et al. [9], the 2.1 Mb chromosome encodes a large proportion of the ABC systems involved in molecular export and cellular process whereas the ABC systems located on the smaller chromosome are largely biased toward nutrient acquisition, leading to the idea that this second chromosome is important in the acquisition and processing of nutrients in *Brucella.*

Since the ABC systems were identified by homology searches, it is possible to assign each ABC importer with a predicted substrate that it imports, providing an overview of the ABC system-based import ability of the *Brucella* species. Table 3 shows the range of predicted substrates imported via ABC transporters within the *Brucella* genomes. Overall, our results show that there is little difference in the import ability between strains of the four species of *Brucella* that are pathogenic to humans (BM, BS, BA, and BC). However, BO lacks the ability to import 8 of the 26 listed nutrients via ABC systems. In fact, all of the 29 pseudogenes that are present within the BO ABC system inventory occur within nutrient importers. The nutrients that BO appears to be unable to import using ABC systems include polyamines (specifically spermidine and putrescine), nickel, thiamine, glycine betaine, erythritol, xylose, and molybdenum. It is possible that the defective uptake of one or more of these substrates by *B. ovis *may contribute to its likely lack of virulence in humans. For example, polyamines have recently been associated with bacterial virulence and pathogenicity in human pathogens [40] and polyamine transporters have therefore been targeted as novel vaccine candidate targets for human pathogens [41, 42]. 

Two predicted erythritol transport systems have been reported that have yet to be confirmed by experimental data [8, 43]. Although the erythritol transporter identified in this study has also been identified by Crasta et al. [43], it should be noted that *B. abortus* S19 has this transport system inactivated by pseudogenes and yet it is still able to incorporate erythritol [43], indicating that this ABC system might not be wholly responsible for erythritol transport. Another study has demonstrated that *B. ovis* does not utilise erythritol as readily as other sugars [44]. 

In this study we have identified one ABC system in BM that we have categorised within a new ABC system family (currently labelled NEW1; See Table 1). This system includes BP and IM proteins related to those of the MOS family and ABC proteins that are different to those from the MOS family. We previously identified a similar ABC system in the genomes of *Burkholderia pseudomallei *and *Burkholderia mallei *strains [45]. Clearly, experimental data is required to define the function of this system.

## 5. Differences between *Brucella* Species

Although there is similarity between the ABC system inventories of the *Brucella* strains studied in this work, we have identified systems that are absent in one or several *Brucella* species (Table 4). The systems that are absent from species are not critical for bacterial survival but could contribute to differences in the lifestyles or virulence of the *Brucella* species. Our data shows that there are ABC systems absent from all of the *Brucella* strains studied. In particular, BO (5 systems), BC (4 systems), and BA (4 systems) lack systems that are present in BM and/or BS. The absence of the ISB (formally ABCX) system from BO and BC is an interesting observation since the ISB systems are soluble complexes involved in labile [Fe-S] biogenesis, which is important in resistance to oxidative stresses. This could indicate that *B. ovis* and *B. canis* reside in environments that are low in oxygen or high in oxygen reducatants, or that they lack enzymes that need labile [Fe-S] centres [46, 47]. Furthermore, this difference may be a factor contributing to the reduced virulence for humans of *B. ovis* and *B. canis* when compared to *B. melitensis, B. suis, *and *B. abortus*. The CDI system absent from *B.ovis * is comprised of two proteins, FtsE (ABC protein) and FtsX (IM protein) [48], and has been studied in *E. coli* and other bacteria including *Bacillus subtilis * [49] and *Mycobacterium tuberculosis* [50]. This CDI system is involved in cell division. *E. coli* mutants of *ftsE* show a reduced growth capacity [51]. The MKL system absent from BC may play a role in toluene tolerance, since Tn5 insertions within the *ttgA2 *gene coding for the MKL ABC protein in *Pseudomonas putida* elicited a toluene-sensitive phenotype [52]. 

## 6. Conclusions

In this study the ABC systems of *B. melitensis *strain 16 M, *B. suis *strain 1330, *B. abortus *9-941, *B. canis *strain RM6/66, and *B. ovis *strain 63/290 have been reannotated using the ABCISSE database in order to provide a new and improved set of annotated *Brucella* ABC systems for the strains studied. The information obtained and the uniform annotation and classification of ABC systems in these closely related species has enabled a more detailed analysis of the roles of ABC systems in *Brucella* species, contributing to an improved understanding of *Brucella* lifestyle and pathogenicity. Previous analysis of the *Brucella* genomes has shown that there is over 90% genome similarity between the *Brucella* species [13, 14]. Similarly, the ABC system inventory compiled in this work reflects the close similarities of the *Brucella* species. However, despite the high genetic homology of *Brucella,* this work highlighted differences in the predicted numbers and functions of the ABC systems encoded by each *Brucella* species. It is widely accepted that the three species that may cause the most human brucellosis are *B. melitensis*, *B. suis,* and *B. abortus *(and occasionally *B. canis)*. This study has shown that these four species of *Brucella* have a larger set of ABC systems encoded within their genomes than *B. ovis,* which is not known to cause human disease. Although it is difficult to ascertain the exact effect of the loss of these ABC systems on *B. ovis*, it is possible to hypothesise that, along with other genetic differences observed [15], they contribute to its overall reduced virulence in humans. It should also be noted there that four further *Brucella* strains have been genome sequenced since this work was completed: *B. melitensis *63/9*, B. abortus *2308*, B. abortus *S19, and *B. suis* Thomsen. Compiling ABC systems inventories of these strains may identify further differences between strains that may have biological relevance. Among the newly sequenced strains are *B. suis* Thomsen, a strain which is not known to cause disease in humans, and *B. abortus* S19, a vaccine strain. ABC system inventories of these strains would be of particular interest since they are considered less pathogenic than the wild-type strains and yet the reasons for this lack of pathogenicity are currently unknown. Overall, the identified differences observed in the ABC system inventory of the *Brucella *strains studied should contribute to a greater understanding of differences in the lifestyles of the *Brucella* species.

## Figures and Tables

**Table 1:** Reconstruction and comparative inventories of *Brucella* ABC systems.

Number	Family	Subfamily	Substrate/Function	Type	*B. melitensis*	*B. abortus*	*B.suis*	*B. ovis*	*B. canis*
1	ART	REG	Involved in gene expression regulation	ABC2	BMEI0288	BruAb11738	BR1753	BOV_1692	BCAN_A1791

2	ART	REG	Involved in gene expression regulation	ABC2	BMEI0553	BruAb11451	BR1456	BOV_1411	BCAN_A1491

3	ART	REG	Involved in gene expression regulation	ABC2	BMEI1258	BruAb10711	BR0692	BOV_0683	BCAN_A0704

4	CBY	CBU	Cobalt import	ABC	**BMEI0635**	BruAb11365	BR1368	BOV_1324	BCAN_A1395
	CBY	CBU	Cobalt import	IM	BMEI0637	BruAb11364	BR1367	BOV_1323	BCAN_A1394, CbiQ

5	CCM		Possibly heme export	IM	BMEI1851		BR0096, ccmC	BOV_0094	BCAN_A0098, ccmC
	CCM		Possibly heme export	IM	BMEI1852		BR0095, ccmB	BOV_0093	BCAN_A0097, ccmB
	CCM		Possibly heme export	ABC	BMEI1853		BR0094, ccmA	BOV_0092	BCAN_A0096, ccmA

6	CDI		Involved in cell division	IM	BMEI0073, ftsX	BruAb11971	BR1996		BCAN_A2042
	CDI		Involved in cell division	ABC	BMEI0072, ftsE	BruAb11972, ftsE	BR1997, ftsE		BCAN_A2043, ftsE

7	CLS		O antigen export system	ABC	BMEI1416, rfbB		BR0519, rfbE	BOV_0523	BCAN_A0531, rfbB
	CLS		O antigen export system	IM	BMEI1415, rfbD		BR0520, rfbD	BOV_0524	BCAN_A0532, rfbD

8	DLM (ABCY)		D-L-Methionine and derivatives import	LPP	BMEI1954				

9	DLM (ABCY)		D-L-Methionine and derivatives import	IM	BMEII0336	BruAb20271	BRA0962	BOV_A0903	BCAN_B0983
	DLM (ABCY)		D-L-Methionine and derivatives import	ABC	BMEII0337	BruAb20272	BRA0961	BOV_A0902	BCAN_B0982
	DLM (ABCY)		D-L-Methionine and derivatives import	LPP	BMEII0338	BruAb20273	BRA0960		

10	DPL	CYD	Cytochrome bd biogenesis and cysteine export	IM-ABC	BMEII0761, cydC	BruAb20713	BRA0509	BOV_A0443	BCAN_B0508
	DPL	CYD	Cytochrome bd biogenesis and cysteine export	IM-ABC	BMEII0762, cydD	BruAb20714, cydD	BRA0508, cydD	BOV_A0442	BCAN_B0507, CydD

11	DPL	MDL	Involved in mitochondrial export systems	IM-ABC	BMEI0323, msbA	BruAb11700	BR1715	BOV_1657	BCAN_A1753

12	DPL	HMT	Involved in mitochondrial export systems	IM-ABC	BMEI0472	BruAb11533	**BR1545**	BOV_1493	BCAN_A1581
	DPL	HMT	Involved in mitochondrial export systems	IM-ABC	BMEI0471	BruAb11534	**BR1544**	BOV_1494	BCAN_A1582

13	DPL	PRT	Proteases, lipase, S-layer protein export	OMP	BMEI1029, TolC	BruAb10954			BCAN_A0957

14	DPL	CHV	Beta-(1–>2) glucan export	IM-ABC	BMEI0984	BruAb11004	BR0998		BCAN_A1015

15	DPL	HMT	Heavy metal tolerance protein	IM-ABC	BMEI1492	BruAb10321	BR0442	BOV_0449	BCAN_A0446

16	DPL	HMT	Involved in mitochondrial export systems	IM-ABC	BMEI1743				
	DPL	HMT	Involved in mitochondrial export systems	IM-ABC	BMEI1742			BOV_0198	

17	DPL	LIP	Involved in lipid A or polysaccharide export	IM-ABC	BMEII0250	BruAb20990	BRA1050	BOV_A0988	BCAN_B1071

18	DRI	YHIH	Unknown	IM	BMEI0656	BruAb11347	BR1349	BOV_1307	BCAN_A1377
	DRI	YHIH	Unknown	IM	BMEI0655				
	DRI	YHIH	Unknown	ABC2	BMEI0654	**BruAb11348**	BR1350 (ABC2-IM)	BOV_1308	**BCAN_A1378**
	DRI	YHIH	Unknown	MFP	BMEI0653	BruAb11349	BR1351	BOV_1309	BCAN_A1379

19	DRI	YHIH	Unknown	IM	BMEII0801	BruAb20757	BRA0465		BCAN_B0467
	DRI	YHIH	Unknown	ABC	BMEII0802, drrA	**BruAb20758**	**BRA0464**	BOV_A0403	**BCAN_B0466**
	DRI	YHIH	Unknown	MFP	BMEII0803	BruAb20759	BRA0463	BOV_A0404	BCAN_B0465

20	DRI	NOS	Nitrous oxide reduction	IM	BMEII0970, nosY	BruAb20902, nosY	BRA0278, nosY	BOV_A0254	BCAN_B0280
	DRI	NOS	Nitrous oxide reduction	ABC	BMEII0971, nosF	BruAb20903, nosF	BRA0277, nosF	BOV_A0253	BCAN_B0279
	DRI	NOS	Nitrous oxide reduction	SS	BMEII0972	BruAb20904, nosD	BRA0276, nosD	BOV_A0252	BCAN_B0278

21	FAE		Fatty acid export	IM-ABC	BMEI0520	BruAb11484	BR1490		BCAN_A1528

22	FAE		Fatty acid export	IM-ABC	BMEII0976	BruAb20908		BOV_A0247	BCAN_B0273

23	HAA		Branched-chain amino acids	IM	BMIE0258, LivH	BruAb11771	BR1790	BOV_1725	BCAN_A1829
	HAA		Branched-chain amino acids	IM	BMIE0259, LivM	BruAb11772	BR1791	BOV_1724	BCAN_A1828
	HAA		Branched-chain amino acids	ABC	BMEI0260, braF	**BruAb11770**	**BR1788**	BOV_1723	BCAN_A1827
	HAA		Branched-chain amino acids	ABC	BMEI0261, braG	BruAb11769	BR1789	BOV_1722	**BCAN_A1826**
	HAA		Branched-chain amino acids	BP	BMEI0263	BruAb11765	BR1785	BOV_1720	BCAN_A1823
	HAA		Branched-chain amino acids	BP	BMEI0264	BruAb11767	BR1782		BCAN_A1820
	HAA		Branched-chain amino acids	BP	BMEI0265			**BOV_1719**	

24	HAA		Branched-chain amino acids	BP	BMEI1930		BR0014	BOV_0012	BCAN_A0014

25	HAA		Branched-chain amino acids	ABC	BMEII0065, livF	BruAb20027	BRA0028	BOV_A0025	BCAN_B0030
	HAA		Branched-chain amino acids	ABC	BMEII0066, livG	BruAb20028	BRA0027	BOV_A0024	**BCAN_B0029 **
	HAA		Branched-chain amino acids	IM	BMEII0067, livM	BruAb20025	BRA0026	BOV_A0023	BCAN_B0028
	HAA		Branched-chain amino acids	IM	BMEII0068, livH	BruAb20026	BRA0025	**BOV_A0022**	BCAN_B0027
	HAA		Branched-chain amino acids	BP	**BMEII0069**	BruAb20024	BRA0024	BOV_A0021	BCAN_B0026

26	HAA		Branched-chain amino acids	ABC	BMEII0098	BruAb21132	BRA1197	BOV_A1099	BCAN_B1227
	HAA		Branched-chain amino acids	ABC	**BMEII0099**	BruAb21133	BRA1196	BOV_A1098	BCAN_B1226
	HAA		Branched-chain amino acids	IM	BMEII0101	BruAb21131	BRA1194	BOV_A1097	**BCAN_B1225 **
	HAA		Branched-chain amino acids	IM	BMEII0102	**BruAb21130**	**BRA1195**	BOV_A1096	BCAN_B1224
	HAA		Branched-chain amino acids	BP	BMEII0103	BruAb21129	BRA1193	BOV_A0195	BCAN_B1223

27	HAA		Branched-chain amino acids	ABC	BMEII0119	BruAb21111	BRA1176	**BOV_A1079**	BCAN_B1207
	HAA		Branched-chain amino acids	IM-ABC	BMEII0120	BruAb21112	BRA1175	BOV_A1078	BCAN_B1206
	HAA		Branched-chain amino acids	IM	BMEII0121	BruAb21110	BRA1174		BCAN_B1205
	HAA		Branched-chain amino acids	BP	BMEII0122	BruAb21109	BRA1173	BOV_A1076	BCAN_B1204

28	HAA		Branched-chain amino acids	IM	BMEII0340	BruAb20276	BRA0957		BCAN_B0977
	HAA		Branched-chain amino acids	IM	BMEII0341	BruAb20277	BRA0956	BOV_A0897	BCAN_B0978
	HAA		Branched-chain amino acids	ABC	BMEII0342	BruAb20278	BRA0955	BOV_A0896	BCAN_B0976
	HAA		Branched-chain amino acids	ABC	BMEII0343	BruAb20279	BRA0954	BOV_A0895	BCAN_B0975
	HAA		Branched-chain amino acids	BP	BMEII0344	BruAb20280	BRA0953	BOV_A0894	BCAN_B0974

29	HAA		Branched-chain amino acids	ABC	BMEII0628	BruAb20574	BRA0652	BOV_A0613	BCAN_B0652
	HAA		Branched-chain amino acids	ABC	BMEII0629	BruAb20575	BRA0651	BOV_A0614	BCAN_B0651
	HAA		Branched-chain amino acids	IM	BMEII0630	BruAb20577	BRA0650	**BOV_A0611**	BCAN_B0649
	HAA		Branched-chain amino acids	IM	BMEII0632	**BruAb20576**	BRA0649	BOV_A0612	BCAN_B0650
	HAA		Branched-chain amino acids	BP	BMEII0633	BruAb20578	BRA0648	BOV_A0610	BCAN_B0648

30	HAA		Branched-chain amino acids	BP	BMEII0875	BruAb20801	BRA0392		BCAN_B0398
	HAA		Branched-chain amino acids	BP	BMEII0868	BruAb20809	BRA0400	**BOV_A0343**	BCAN_B0395
	HAA		Branched-chain amino acids	ABC	BMEII0874	BruAb20806	BRA0395	BOV_A0338	BCAN_B0389
	HAA		Branched-chain amino acids	ABC	BMEII0873	BruAb20807	**BRA0394**	BOV_A0337	BCAN_B0396
	HAA		Branched-chain amino acids	IM		BruAb20808	BRA0393	BOV_A0336	**BCAN_B0397**

31	ISB (ABCX)		Iron/sulphur centre biogenesis	CYTP	BMEI1040	BruAb10941	BR0931		
	ISB (ABCX)		Iron/sulphur centre biogenesis	CYTP	BMEI1042	BruAb10940	BR0933		
	ISB (ABCX)		Iron/sulphur centre biogenesis	ABC	BMEI1041	BruAb10942	BR0932		

32	ISVH		Iron-siderophores, VB12 and Hemin import	ABC	BMEI0660	BruAb11342	BR1344	BOV_1302	BCAN_A1371
	ISVH		Iron-siderophores, VB12 and Hemin import	IM	BMEI0659	BruAb11343	BR1345	BOV_1304	BCAN_A1372
	ISVH		Iron-siderophores, VB12 and Hemin import	OMR	BMEI0657	BruAb11344	BR1347	BOV_1306	BCAN_A1374
	ISVH		Iron-siderophores, VB12 and Hemin import	BP	BMEI0658	BruAb11345	BR1346	BOV_1305	BCAN_A1373

33	ISVH		Iron(III) dicitrate import	BP	BMEII0535	BruAb20476	BRA0756	BOV_A0705	BCAN_B0763
	ISVH		Iron(III) dicitrate import	IM	BMEII0536, fecD	BruAb20477	BRA0755	BOV_A0704	BCAN_B0764
	ISVH		Iron(III) dicitrate import	ABC	BMEII0537, fecE	BruAb20478	BRA0754	BOV_A0703	BCAN_B0762

34	ISVH		Iron(III) import	ABC	BMEII0604	BruAb20550	BRA0678	BOV_A0635	BCAN_B0677
	ISVH		Iron(III) import	IM	BMEII0605, fatC	BruAb20551	BRA0676	BOV_A0634	BCAN_B0675
	ISVH		Iron(III) import	IM	BMEII0606, fatD	BruAb20552	BRA0677	BOV_A0633	BCAN_B0676
	ISVH		Iron(III) import	BP	BMEII0607	BruAb20553	BRA0675	BOV_A0632	BCAN_B0674

35	MET		Zinc import	IM	BMEII0176, ZnuB	BruAb21061, ZnuB	BRA1124, ZnuB	BOV_A1029	BCAN_B1152
	MET		Zinc import	ABC	BMEII0177, ZnuC	BruAb21060, ZnuC	BRA1123, ZnuC	BOV_A1028	BCAN_B1151
	MET		Zinc import	BP	BMEII0178, ZnuA	BruAb21059, ZnuA	BRA1122, ZnuA	BOV_A1027	BCAN_B1150

36	MKL		Involved in toluene tolerance	ABC	BMEI0964	BruAb11025	BR1020	BOV_0987	
	MKL		Involved in toluene tolerance	IM	BMEI0965, ttg2B	BruAb1024	BR1019	BOV_0986	
	MKL		Involved in toluene tolerance	SS	BMEI0963, ttg2C	BruAb11026	BR1021	BOV_0988	

37	MOI		Thiamine import	ABC	BMEI0283, thiQ	BruAb11744	BR1759	BOV_1698	BCAN_A1798
	MOI		Thiamine import	IM	BMEI0284, thiP	BruAb11743, thiP	BR1758, thiP	**BOV_1696**	thiP, BCAN_A1797
	MOI		Thiamine import	BP	BMEI0285	BruAb11744, thiB	BR1757, thiB	BOV_1695	thiB, BCAN_A1796
38	MOI		Putrescine import	BP	BMEI0411, potF	BruAb11599	BR1612	BOV_1556	BCAN_A1649
	MOI		Putrescine import	ABC	BMEI0412	BruAb11598	BR1611	**BOV_1555**	BCAN_A1648
	MOI		Putrescine import	IM	BMEI0413	BruAb11596	BR1609	**BOV_1554**	BCAN_A1647
	MOI		Putrescine import	IM	BMEI0414	**BruAb11597**	BR1610	**BOV_1553**	BCAN_A1646

39	MOI		Sulphate import	IM	BMEI0675, cysW	BruAb11328, cysW2	BR1328, cysW2	BOV_1288	CysW, BCAN_A1353
	MOI		Sulphate import	IM	BMEI0674, cysT	BruAb11329	BR1329	BOV_1289	CysT, BCAN_A1354
	MOI		Sulphate import	BP	BMEI0673	BruAb11330	BR1330	BOV_1290	BCAN_A1355

40	MOI		Sulphate import	ABC	BMEI1838 cysA	BruAb10107	BR0110	BOV_0107	CysA, BCAN_A0113
	MOI		Sulphate import	IM	BMEI1839, cysW	BruAb10106	BR0109, cysW1	BOV_0106	CysW, BCAN_A0112
	MOI		Sulphate import	IM	BMEI1840, cyst	BruAb10105, cysT	BR0108	BOV_0105	CysT, BCAN_A0111
	MOI		Sulphate import	BP	BMEI1841	BruAb10104	BR0107	BOV_0104	BCAN_A0110

41	MOI		Phosphate import	ABC	BMEI1986, pstB	BruAb12116, pstB	BR2141, pstB	BOV_2056	BCAN_A2185, pstB
	MOI		Phosphate import	IM	BMEI1987, pstA	BruAb12114, pstC	BR2139, pstC	BOV_2055	BCAN_A2184, pstA
	MOI		Phosphate import	IM	BMEI1988, pstC	BruAb12115, pstA	BR2140	BOV_2054	BCAN_A2183, pstC
	MOI		Phosphate import	BP	BMEI1989	BruAb12113	BR2138	BOV_2053	BCAN_A2128

42	MOI		Molybdenum import	ABC	BMEII0003, modC	BruAb20090	BRA0090, modC	**BOV_A0084**	BCAN_B0093, ModC
	MOI		Molybdenum import	IM	BMEII004, modB	BruAb20089	BRA0089, modB	BOV_A0083	BCAN_B0092, ModB
	MOI		Molybdenum import	BP	BMEII0005	BruAb20088	BRA0088, modA	BOV_A0082	BCAN_B0091

43	MOI		Spermidine/putrescine import	ABC	BMEII0193, potA	BruAb21046	**BRA1107**		**BCAN_B1129**
	MOI		Spermidine/putrescine import	IM	BMEII0194, potB	BruAb21044	BRA1106		BCAN_B1128
	MOI		Spermidine/putrescine import	IM	BMEII0195, potC	BruAb21045	BRA1105		BCAN_B1127
	MOI		Spermidine/putrescine import	BP	BMEII0196	BruAb21043	**BRA1104**		BCAN_B1126

44	MOI		Unknown	BP	BMEII0479	BruAb20422	BRA0810	BOV_A0760	BCAN_B0824
	MOI		Unknown	ABC	BMEII0481	BruAb20423	BRA0809	BOV_A0759	BCAN_B0823
	MOI		Unknown	IM	BMEII0483	BruAb20424	BRA0807	BOV_A0758	BCAN_B0822
	MOI		Unknown	IM	BMEII0484	BruAb20425	**BRA0808**	BOV_A0757	BCAN_B0821

45	MOI		Iron(III) import	BP	BMEII0565	BruAb20510	BRA0720	BOV_A0676	BCAN_B0726
	MOI		Iron(III) import	IM2	BMEII0566	BruAb20511	BRA0719	BOV_A0675	BCAN_B0274
	MOI		Iron(III) import	ABC	BMEII0567	BruAb20512	BRA0718	BOV_A0674	BCAN_B0725

46	MOI		Iron(III) import	ABC	BMEII0583	BruAb20529	BRA0701	BOV_A0656	BCAN_B0702
	MOI		Iron(III) import	BP	BMEII0584	BruAb20530	BRA0700	BOV_A0655	BCAN_B0703
	MOI		Iron(III) import	IM2	BMEII0585	BruAb20531	BRA0699	BOV_A0654	BCAN_B0701

47	MOI		Spermidine/putrescine import	IM	BMEII0920, potC	BruAb20852	BRA0328	BOV_A0303	BCAN_B0331
	MOI		Spermidine/putrescine import	IM	BMEII0921, potB	BruAb20853	BRA0329	**BOV_A0302**	BCAN_B0330
	MOI		Spermidine/putrescine import	ABC	BMEII0922, potA	BruAb20855	BRA0327	BOV_A0301	BCAN_B0329
	MOI		Spermidine/putrescine import	BP	BMEII0923, potD	BruAb20854	BRA0326	BOV_A0300	BCAN_B0328

48	MOI		Iron(III) import	BP	BMEII1120	BruAb20113	BRA0115	BOV_A0105	BCAN_B0119
	MOI		Iron(III) import	IM	BMEII1121, sufB	BruAb20111	BRA0114	BOV_A0104	BCAN_B0118
	MOI		Iron(III) import	IM	BMEII1122, sufB	BruAb20112	BRA0113	BOV_A0103	BCAN_B0117
	MOI		Iron(III) import	ABC	BMEII1123, sufC	BruAb20110	BRA0112	BOV_A0102	BCAN_B0116

49	New1		Unknown	IM	BMEI0013	BruAb12030	BR2055	BOV_1975	BCAN_A2101
	New1		Unknown	ABC	BMEI0012	BruAb12031	BR2056		BCAN_A2102
	New1		Unknown	BP	BMEI0014				
	New1		Unknown	BP	BMEI0015				

50	MOS		Ribose import	ABC2	BMEI0391	BruAB11620, rbsA-2	BR1632, rbsA-2	BOV_1576	BCAN_A1669
	MOS		Ribose import	IM	BMEI0392	BruAB11619, rbsC-2	BR1631, rbsC-2	BOV_1575, rbsC2	BCAN_A1668
	MOS		Ribose import	BP	BMEI0393	BruAB11618	BR1630	BOV_1574	BCAN_A1667

51	MOS		Ribose Import	ABC	BMEI0665	BruAb11337	BR1339	**BOV_1299**	BCAN_A1367
	MOS		Ribose Import	IM	BMEI0664	**BruAb11338**	BR1340	BOV_1300	BCAN_A1368
	MOS		Ribose Import	BP	BMEI0663	BruAb11340	BR1342	**BOV_1301**	BCAN_A1369
	MOS		Ribose Import	BP	BMEI0662	BruAb11335			

52	MOS		Ribose import	BP	BMEI1390	BruAb10566, rbsB1	BR0544, rbsB1	BOV_0546 rbsB1	BCAN_A0557
	MOS		Ribose import	IM	BMEI1391, rbsC	BruAb10565, rbsC1	BR0543, rbsC1	BOV_0545 rbsC1	BCAN_A0555
	MOS		Ribose import	ABC2	BMEI1392, rbsA	BruAb10564, rbsA1	BR0542, rbsA1	BOV_0544 rbsA1	BCAN_A0554, rsbA

53	MOS		Possibly galactoside	BP	**BMEII0083**	BruAb20010	BRA0010	BOV_A0007	
	MOS		Possibly galactoside	ABC2	BMEII0085, mglA	BruAb20009	BRA0009	BOV_A0006	
	MOS		Possibly galactoside	IM	BMEII0086, mglC	BruAb20007	BRA0007	BOV_A0005	
	MOS		Possibly galactoside	IM	BMEII0087	BruAb20008	BRA0008	BOV_A0004	

54	MOS		Xylose import	IM	BMEII0144, xylH	BruAb21089, xylH	BRA1152, xylH	BOV_A1057	BCAN_B1181
	MOS		Xylose import	ABC2	BMEII0145, xylG	BruAb21088, xylG	BRA1151, xylG	**BOV_A1056**	BCAN_B1180, xylG
	MOS		Xylose import	BP	BMEII0146, xylF	BruAb21087, xylF	BRA1150, xylF	**BOV_A1055**	BCAN_B1179, xylF

55	MOS		Ribose import	ABC2	BMEII0300, rbsA	BruAb20239rbsA4	BRA0995, rbsA4	**BOV_A0937**	BCAN_B1014
	MOS		Ribose import	IM	BMEII0301 rbsC	BruAb20240,rbsC5	BRA0993, rbsC5		BCAN_B1013
	MOS		Ribose import	IM	BMEII0302 rbsC	BruAb20239, rbsC4	BRA0994, rbsC5	BOV_A0935	BCAN_B1012
	MOS		Ribose import	BP		**BruAb20238**	BRA0996, rbsB3	BOV_A0938	BCAN_B1015

56	MOS		Monosaccharide import	BP	**BMEII0360, chvE**	**BruAb20296**	BRA0937	BOV_A0879	BCAN_B0957
	MOS		Monosaccharide import	ABC2	BMEII0361	BruAb20297	BRA0936	BOV_A0878	BCAN_B0956
	MOS		Monosaccharide import	IM	BMEII0362	BruAb20298	BRA0935	BOV_A0877	BCAN_B0955

57	MOS		Erythritol import	ABC2	BMEII0432, rbsA	BruAb20371, rbsA3	BRA0860, rbsA3	BOV_A0807, rsbA3	BCAN_B0877
	MOS		Erythritol import	IM	BMEII0433, rbsC	BruAb20372, rbsC3	BRA0859, rbsC3		BCAN_B0876
	MOS		Erythritol import	BP	BMEII0435	BruAb20373, rbsB2	BRA0858, rbsB2	**BOV_A0805**	BCAN_B0875

58	MOS		Galactoside/Ribose import	ABC2	**BMEII0698**	BruAb20654	BRA0570	BOV_A0533	BCAN_B0570
	MOS		Galactoside/Ribose import	IM				BOV_A0534	BCAN_B0567
	MOS		Galactoside/Ribose import	IM	BMEII0700	BruAb20655	BRA0568	BOV_A0535	
	MOS		Galactoside/Ribose import	IM	BMEII0701	BruAb20656	BRA0569		BCAN_B0568
	MOS		Galactoside/Ribose import	BP	BMEII0702		BRA0567	BOV_A0532	BCAN_B0567

59	MOS		Monosaccharide import	IM	BMEII0981	BruAb20913	BRA0267	BOV_A0242	BCAN_B0268
	MOS		Monosaccharide import	ABC2	BMEII0982	BruAb20914	BRA0266	BOV_A0241	BCAN_B0267
	MOS		Monosaccharide import	BP	BMEII0983	BruAb20916	BRA0265	BOV_A0240	BCAN_B0266

60	o228		Unknown	IM	BMEI0361				
	O228		Unknown	MFP	BMEI0359				
	o228		Unknown	ABC	BMEI0360				

61	o228		Unknown	IM		BruAb10085	BR0087	BOV_0085	
	O228		Unknown	MFP					BCAN_A1712
	o228		Unknown	ABC		BruAb10084	BR0086	BOV_0084	BCAN_A1711

62	o228		Unknown	MFP		**BruA11658**	**BR1671**	BOV_1617	
	o228		Unknown	IM-ABC		**BruA11657**	**BR1670**	BOV_1616	BCAN_A0087

63	o228		Lipoprotein release system	ABC	BMEI1138, LolD	BruAb10838,LolD	BR0824, LolD	BOV_0818	BCAN_A0839
	o228		Lipoprotein release system	IM	BMEI1139, LolE	BruAb10837, LolE	BR0823, LolE	BOV_0817	BCAN_A0838

64	OPN		Dipeptide import	ABC	BMEI0438, dppF	BruAb11569	BR1582	**BOV_1527**	BCAN_A1617
	OPN		Dipeptide import	ABC	BMEI0437, dppD	BruAb11570	BR1583	BOV_1528	BCAN_A1618
	OPN		Dipeptide import	IM	BMEI0435, dppC	BruAb11571	BR1584	BOV_1530	BCAN_A1620
	OPN		Dipeptide import	IM	BMEI0436, dppC	BruAb11572	BR1585	BOV_1529	BCAN_A1619
	OPN		Dipeptide import	BP	**BMEI0433, dppA**	**BruAb11573**	**BR1586**	**BOV_1531**	**BCAN_A1621**

65	OPN		Oligopeptide import	ABC2	BMEI1938, oppD	BruAb10006	BR0006	BOV_0006	BCAN_A0006
	OPN		Oligopeptide import	BP	BMEI1934	BruAb10007	BR0007	BOV_0009	BCAN_A0010
	OPN		Oligopeptide import	BP	BMEI1935	BruAb10008	BR0008	BOV_0010	BCAN_A0009
	OPN		Oligopeptide import	IM	BMEI1936, oppB	BruAb10009	BR0009	BOV_0008	BCAN_A0008
	OPN		Oligopeptide import	IM	BMEI1937, oppC	BruAb10010	BR0010	BOV_0007	BCAN_A0007

66	OPN		Oligopeptide import	ABC	BMEII0199, oppF	BruAb21039	**BRA1100**		
	OPN		Oligopeptide import	ABC	BMEII0200, oppD	BruAb21040	BRA1101		**BCAN_B1123 **
	OPN		Oligopeptide import	IM	BMEII0201, oppC	BruAb21037	BRA0099		BCAN_B1122
	OPN		Oligopeptide import	IM	BMEII0202, oppB	BruAb21038	BRA0098		BCAN_B1121
	OPN		Oligopeptide import	BP	BMEII01203	BruAb21036	BRA0097		**BCAN_B1119**

67	OPN		Dipeptide import	ABC	BMEII0205, dppF	BruAb21033	**BRA1095**	BOV_A0950	BCAN_B1117
	OPN		Dipeptide import	ABC	BMEII0206, dppD	BruAb21034	BRA1094	BOV_A0951	BCAN_B1116
	OPN		Dipeptide import	IM	**BMEII0207, dppC**	BruAb21031	BRA1093		BCAN_B1115, dppC
	OPN		Dipeptide import	IM		BruAb21032	BRA1092	BOV_A0952	
	OPN		Dipeptide import	IM	BMEII0209, dppB			BOV_A0953	BCAN_B1114
	OPN		Dipeptide import	BP	BMEII0210	**BruAb21030**	BRA1090	BOV_A0954	BCAN_B1113

68	OPN		Dipeptide/ Oligopeptide import	BP	BMEII0217	BruAb21024	BRA1084		BCAN_B1107
	OPN		Dipeptide/ Oligopeptide import	IM	BMEII0220	BruAb21020	BRA1081		BCAN_B1104
	OPN		Dipeptide/ Oligopeptide import	IM	BMEII0221	BruAb21021	BRA1080		BCAN_B1103
	OPN		Dipeptide/ Oligopeptide import	ABC	BMEII0222	BruAb21018	BRA1079		BCAN_B1102
	OPN		Dipeptide/ Oligopeptide import	ABC	BMEII0223	BruAb21019	BRA1078		BCAN_B1101

69	OPN		Dipeptide import	BP	BMEII0284	BruAb20952	BRA1012	BOV_A0504	BCAN_B1032
	OPN		Dipeptide import	IM	BMEII0285	BruAb20950	BRA1009	BOV_A0501	BCAN_B1031
	OPN		Dipeptide import	IM	BMEII0286	BruAb20951	BRA1008	BOV_A0502	BCAN_B1030
	OPN		Dipeptide import	ABC	BMEII0287	BruAb20948	BRA1011	BOV_A0500	BCAN_B1029
	OPN		Dipeptide import	ABC	BMEII0288	BruAb20949	BRA1010	BOV_A0501	BCAN_B1028

70	OPN		Nickel import	BP	BMEII0487	**BruAb20428**	BRA0804	BOV_A0754	BCAN_B0818, NikA
	OPN		Nickel import	IM	BMEII0488, nikB	BruAb20429, nikB	BRA0802, nikC	BOV_A0752	BCAN_B0817, NikB
	OPN		Nickel import	IM	BMEII0489, nikC	BruAb20430, nikV	BRA0803, nikB	BOV_A0753	BCAN_B0816, NikC
	OPN		Nickel import	ABC	BMEII0490, nikD	BruAb20431, nikD	BRA0800, nikE	**BOV_A0751**	BCAN_B0815, NikD
	OPN		Nickel import	ABC	BMEII0491, nikE	BruAb20432, nikE	BRA0801, nikD		BCAN_B0814, NikE

71	OPN		Oligopeptide import	BP	BMEII0504	BruAb20446	**BRA0783**	**BOV_A0737**	BCAN_B0800
	OPN		Oligopeptide import	IM	BMEII0505	BruAb20447	BRA0788	BOV_A0736	BCAN_B0799
	OPN		Oligopeptide import	IM	BMEII0506	BruAb20448	BRA0787	BOV_A0735	BCAN_B0798
	OPN		Oligopeptide import	ABC	BMEII0507	**BruAb20449**	BRA0786	BOV_A0734	BCAN_B0797
	OPN		Oligopeptide import	ABC	BMEII0508			**BOV_A0733**	BCAN_B0796

72	OPN		Oligopeptide import	BP	BMEII0691	BruAb20648	BRA0576	BOV_A0542	

73	OPN		Oligopeptide import	BP	BMEII0734	BruAb20684	BRA0538	BOV_A0468	BCAN_B0538
	OPN		Oligopeptide import	BP	BMEII0735, oppA	BruAb20685	BRA0537	BOV_A0467	BCAN_B0537
	OPN		Oligopeptide import	IM	BMEII0736	BruAb20686	BRA0536	BOV_A0466	BCAN_B0535
	OPN		Oligopeptide import	IM	BMEII0737	BruAb20687	BRA0535	BOV_A0465	BCAN_B0536
	OPN		Oligopeptide import	ABC2	BMEII0738	BruAb20688	BRA0534	BOV_A0464	BCAN_B0534

74	OPN		Oligopeptide import	BP	BMEII0859	BruAb20792	BRA0409	BOV_A0352	BCAN_B0412
	OPN		Oligopeptide import	IM	BMEII0860		BRA0408	BOV_A0351	BCAN_B0411
	OPN		Oligopeptide import	IM	BMEII0861	BruAb20794	BRA0407	BOV_A0350	BCAN_B0410
	OPN		Oligopeptide import	ABC	BMEII0863	BruAb20796	BRA0405	BOV_A0347	BCAN_B0408
	OPN		Oligopeptide import	ABC	BMEII0864	BruAb20797	BRA0404	BOV_A0348	BCAN_B0407

75	OSP		Maltose import	ABC	BMEI1713, malK	BruAb10233	BR0238	BOV_0231	BCAN_A0241
	OSP		Maltose import	IM	BMEI1714, malG	BruAb10231	BR0237	BOV_0230	BCAN_A0240
	OSP		Maltose import	IM	BMEI1715, malF	BruAb10232	BR0236	BOV_0229	BCAN_A0239
	OSP		Maltose import	BP	BMEI1716	BruAb10230	BR0235	BOV_0228	BCAN_A0238

76	OSP		Oligosaccharide or polyol import	ABC	BMEII0112, ugpC	BruAb21119	BRA1183	BOV_A1086	BCAN_B1214
	OSP		Oligosaccharide or polyol import	IM	BMEII0113, ugpA	BruAb21118	BRA1181	BOV_A1085	BCAN_B1213
	OSP		Oligosaccharide or polyol import	IM	BMEII0114, ugpE	**BruAb21117**	BRA1182	BOV_A1084	BCAN_B1212
	OSP		Oligosaccharide or polyol import	BP	BMEII0115	BruAb21116	BRA1180		BCAN_B1211
77	OSP		Oligosaccharide or polyol import	IM	BMEII0541	BruAb20483	BRA0749	BOV_A0700	BCAN_B0757
	OSP		Oligosaccharide or polyol import	IM		BruAb20482	BRA0750	BOV_A0699	BCAN_B0756
	OSP		Oligosaccharide or polyol import	BP	BMEII0542	BruAb20484	BRA0748	BOV_A0698	BCAN_B0755
	OSP		Oligosaccharide or polyol import	ABC	BMEII0544	BruAb20487	BRA0745	BOV_A0696	BCAN_B0753

78	OSP		Oligosaccharide or polyol import	BP	BMEII0590	BruAb20537	**BRA0693**	**BOV_A0648**	BCAN_B0691
	OSP		Oligosaccharide or polyol import	IM	BMEII0591	BruAb20538	BRA0691	BOV_A0647	BCAN_B0690
	OSP		Oligosaccharide or polyol import	IM	BMEII0592	**BruAb20539**	BRA0692	BOV_A0646	BCAN_B0689
	OSP		Oligosaccharide or polyol import	ABC	BMEII0593	BruAb20540	BRA0690	BOV_A0645	BCAN_B0688

79	OSP		SN-glycerol-3-phosphate import	ABC	BMEII0621, ugpC	BruAb20568, ugpC	BRA0658, ugpC	BOV_A0620	BCAN_B0658
	OSP		SN-glycerol-3-phosphate import	IM	BMEII0622, ugpE	BruAb20569, ugpE	BRA0657, ugpE	BOV_A0619	BCAN_B0657
	OSP		SN-glycerol-3-phosphate import	IM	BMEII0623, ugpE	BruAb20570, ugpA	BRA0656, ugpA	BOV_A0618	**BCAN_B0656**
	OSP		SN-glycerol-3-phosphate import	IM	BMEII0624, ugpA				
	OSP		SN-glycerol-3-phosphate import	BP	BMEII0625	BruAb20571, ugpB	BRA0655, ugpA	BOV_A0617	BCAN_B0655

80	OSP		Oligosaccharide or polyol import	ABC	BMEII0750	BruAb20702	BRA0521	BOV_A0454	BCAN_B0520
	OSP		Oligosaccharide or polyol import	IM	BMEII0752	BruAb20704	BRA0519	BOV_A0452	BCAN_B0518
	OSP		Oligosaccharide or polyol import	IM	BMEII0753	BruAb20705	BRA0518	BOV_A0451	BCAN_B0517
	OSP		Oligosaccharide or polyol import	BP	BMEII0754	**BruAb20706**	BRA0516	BOV_A0449	BCAN_B0516
	OSP		Oligosaccharide or polyol import	BP	BMEII0755				

81	OSP		Maltose import	ABC	**BMEII0940**	BruAb20874	BRA0307	BOV_A0282	BCAN_B0308
	OSP		Maltose import	IM	BMEII0942	**BruAb20875**	BRA0306	**BOV_A0281**	BCAN_B0307
	OSP		Maltose import	IM	BMEII0943	**BruAb20876**	BRA0305	BOV_A0280	BCAN_B0306
	OSP		Maltose import	BP	BMEII0944			**BOV_A0279**	
	OSP		Maltose import	BP	BMEII0945	BruAb20877	BRA0304		BCAN_B0305

82	OTCN		Glycine betaine/L-proline import	ABC	BMEI0439, proV	BruAb11568	BR1581	BOV_1526	BCAN_A1616
	OTCN		Glycine betaine/L-proline import	IM	BMEI0440, proW	BruAb11567	BR1580	BOV_1525	BCAN_A1615
	OTCN		Glycine betaine/L-proline import	BP	BMEI0441, proX	BruAb11566	BR1579	BOV_1524	BCAN_A1614

83	OTCN		Choline S^S^-dependent regulation of *yehZYXW *	BP	BMEI1725	BruAb10220	BR0225	BOV_0216	BCAN_A0228
	OTCN		Choline S^S^-dependent regulation of *yehZYXW *	IM	BMEI1726, proW	BruAb10217	BR1222	BOV_0215	BCAN_A0227
	OTCN		Choline S^S^-dependent regulation of *yehZYXW *	IM	BMEI1728, proW	BruAb10219	BR0224	BOV_0213	BCAN_A0225
	OTCN		Choline S^S^-dependent regulation of *yehZYXW *	ABC	BMEI1727, proV	BruAb10218	BR0223	BOV_0214	BCAN_A0226

84	OTCN		Osmoprotectants, Taurine, Cyanante & Nitrate	BP	BMEI1737	BruAb10207	BR0211	BOV_0204	BCAN_A0215
	OTCN		Osmoprotectants, Taurine, Cyanante & Nitrate	IM	BMEI1739	BruAb10206	BR0213	BOV_0202	BCAN_A0213
	OTCN		Osmoprotectants, Taurine, Cyanante & Nitrate	ABC		BruAb10208			

85	OTCN		Taurine import	BP	BMEII0109	BruAb21122	BRA1186	BOV_A1089	BCAN_B1218
	OTCN		Taurine import	IM	BMEII0107, tauC	BruAb21124	BRA1188	**BOV_A1091**	BCAN_B1219
	OTCN		Taurine import	ABC	BMEII0108, tauB	BruAb21123	BRA1187	BOV_A1090	BCAN_B1217

86	OTCN		Glycine betaine/L-proline import	ABC	BMEII0548	**BruAb20492**	BRA0740	BOV_A0692	BCAN_B0748
	OTCN		Glycine betaine/L-proline import	IM	BMEII0549	BruAb20493	BRA0739	BOV_A0691	BCAN_B0747
	OTCN		Glycine betaine/L-proline import	BP	BMEII0550	BruAb20494	BRA0738	**BOV_A0690**	BCAN_B0746

87	OTCN		Nitrate import	BP	BMEII0797	BruAb20753	BRA0469	BOV_A0406	BCAN_B0471
	OTCN		Nitrate import	ABC	BMEII0798, nrtC	BruAb20755	BRA0467	BOV_A0407	BCAN_B0470
	OTCN		Nitrate import	IM	BMEII0799, nrtB	BruAb20755	BRA0468	BOV_A0408	BCAN_B0469

88	OTCN		Taurine import	ABC	BMEII0961	BruAb10894	BRA0286	BOV_A0262	BCAN_B0288
	OTCN		Taurine import	IM	BMEII0962	BruAb10895	BRA0285	BOV_A0261	BCAN_B0287
	OTCN		Taurine import	BP	BMEII0963	BruAb10896	BRA0284	**BOV_A0260**	BCAN_B0286

89	PAO		Polar amino acid import	ABC	BMEI0108	BruAb11932	BR1959	BOV_A0336	BCAN_A2004
	PAO		Polar amino acid import	ABC	BMEI0111	BruAb11935	BR1956	BOV_1885	BCAN_A2001
	PAO		Polar amino acid import	IM	BMEI0112	BruAb11931	BR1955	BOV_1882	BCAN_A2000
	PAO		Polar amino acid import	IM	BMEI0113	**BruAb11930**	**BR1954**	BOV_1081	BCAN_A1999
	PAO		Polar amino acid import	BP	**BMEI0114**	BruAb11929	BR1953	BOV_1880	BCAN_A1998
	PAO		Polar amino acid import	BP				BOV_1879	

90	PAO		Arginine/Ornithine biding protein precursor	BP		BruAb20594		BOV_A0594	
	PAO		Arginine/Ornithine biding protein precursor	BP	BMEI1022	BruAb20595	BRA0632	BOV_A0593	
	PAO		Arginine/Ornithine biding protein precursor	BP		BruAb10874	BRA0631	BOV_0945	BCAN_A0967

91	PAO		General L-amino acid import	ABC	BMEI1208, appP	**BruAb10762**	BR0745	BOV_A0890	BCAN_A0760
	PAO		General L-amino acid import	IM	BMEI1209, appM	**BruAb10758**	BR0744	BOV_0739	BCAN_A0759
	PAO		General L-amino acid import	IM	BMEI1210, appQ	**BruAb10760**	BR0743	BOV_0737	BCAN_A0758
	PAO		General L-amino acid import	BP	BMEI1211, appJ	**BruAb10761**	BR0741	BOV_0738	BCAN_A0756
	PAO		General L-amino acid import	BP	BMEII0349, appJ	**BruAb20285**	BRA0948	BOV_0736	BCAN_B0969

92	PAO		Arginine	BP	BMEI1627	BruAb10321	BR0295	BOV_0308	

93	PAO		Cystine import	ABC	BMEII0599	BruAb20545	BRA0684	BOV_A0640	BCAN_B0682
	PAO		Cystine import	IM	BMEII0600	BruAb20546	BRA0683	BOV_A0639	BCAN_B0681
	PAO		Cystine import	BP	BMEII0601	BruAb20547, fliY	BRA0682, fliY	BOV_A0638, fliY	BCAN_B0680

94	PAO		Polar amino acid import	IM			BR0952		BCAN_A0964
	PAO		Polar amino acid import	IM			BR0953		BCAN_A0965
	PAO		Polar amino acid import	BP	BMEI1104		BR0955	BOV_0854	

95	PAO		Polar amino acid import	BP			BR0862	BOV_A0903	

96	UVR		DNA repair	ABC2	BMEI0878	BruAb1110, UvrA	UvrA	BOV_1063	BCAN_A1124
97	YHBG		Possible LPS transport to outer membrane	ABC	BMEI1790	BruAb10153	BR157	BOV_0152	BCAN_A0162
	YHBG		Possible LPS transport to outer membrane	SS	BMEI1791	BruAb10152	BR156	BOV_0151	BCAN_A0161

ABC: ATP-Binding Cassette; IM: Inner membrane protein; BP: Binding protein; IM-ABC: Inner membrane protein-ATP binding cassette fusion; ABC2: 2 ABC proteins fused together; OMP: Outer membrane protein; MFP: Membrane fusion protein; SS: Signal sequence; LPP: Extracytoplasmic protein with a lipoprotein type signal sequence; BM: *Brucella melitensis; *BA: *Brucella abortus; *BS: *Brucella suis; *Bold Text: Indicates a frame shift mutation or premature stop codon in these genes.

**Table 2:** ABC system families/subfamilies.

Name		Description and Function
Family	Subfamily	
Exporters (predicted and experimental)		

DPL, *Drugs, Peptides, *	HMT	Mitochondrial and bacterial transporters II
*Lipids*	CHV	Beta(1–2) Glucan export
	MDL	Mitochondrial and bacterial transporters I
	LIP	Lipid A or glycerophospholipid export
	PRT	Proteases, Lipases, S-Layer protein export
	CYD	Cytochrome bd biogenesis
CCM		Cytochrome C biogenesis
CLS		Capsular polysaccharide, lipopolysaccharide and teichoic acids
FAE		Fatty Acid Export

Importers		

DLM		D- L-Methionine and derivatives
CBY	CBU	Cobalt uptake, putative
MKL		Related to MOI family but unknown substrate
YHBG		Related to HAA family, but unknown substrate
CDI		Cell division
MET		Metals
MOS		Monosaccharides
MOI		Mineral and Organic ions
PAO		Polar amino acids and Opines
HAA		Hydrophobic amino acids and amides
OSP		Oligosaccharides and polyols
OPN		Oligopeptides and Nickel
OTCN		Osmoprotectants Taurine Cyanate and Nitrate
ISVH		Iron-Siderophores VitaminB-12 and Hemin

cellular process (experimental)		

ISB		Iron-sulphur centre biogenesis
ART,* Antibiotic resistance and translation regulation *	REG	Translation regulation
UVR		DNA repair and drug resistance

Unknown		

DRI, *Drug resistance, bacteriocin, and lantibiotic immunity *	YHIH	Drug resistance, putative
	NOS	Possible nitrous oxide reduction
NO		Unclassified Systems
o228		Unknown

**Table 3:** *Brucella* ABC import ability.

Substrate	*B. melitensis*	*B. abortus*	*B. suis*	*B. ovis*	*B. canis*
Branch chain amino acids	∗∗∗∗	∗∗∗	∗∗∗	∗∗	∗∗∗
Iron (III)	∗∗∗∗	∗∗∗∗	∗∗∗∗	∗∗∗∗	∗∗∗∗
Cobalt	—	∗	∗	∗	∗
Zinc	∗	∗	∗	∗	∗
Thiamine	∗	∗	∗	—	∗
Putrescine	∗∗∗	∗∗	∗∗	—	∗∗
Sulphate	∗∗	∗∗	∗∗	∗∗	∗∗
Phosphate	∗	∗	∗	∗	∗
Molybdenum	∗	∗	∗	—	∗
Spermidine	∗∗	∗∗	∗	—	∗
Ribose	∗∗∗	∗∗∗	∗∗∗	∗∗∗	∗∗∗
Galactoside	—	∗∗	∗∗	∗∗	∗
Xylose	∗	∗	∗	—	∗
Erythritol	∗	∗	∗	—	∗
Dipeptides	∗∗	∗∗	∗∗	∗∗	∗∗∗
Oligopeptides	∗∗∗∗	∗∗∗∗	∗∗∗	∗∗∗	∗∗∗∗
Nickel	∗	—	∗	—	∗
Maltose	∗	∗	∗	∗	∗
Oligosaccharide or polyol	∗∗∗	∗	∗∗	∗∗	∗∗∗
SN-glycerol-3-phosphate	∗	∗	∗	∗	—
Taurine	∗∗∗	∗∗∗	∗∗∗	∗	∗∗∗
Glycine betaine	∗	—	∗	—	∗
Nitrate	∗	∗	∗	∗	∗
Polar amino acids	—	—	—	∗	∗
Cystine	∗	∗	∗	∗	∗
General L amino acids	∗	—	∗	∗	∗

This table does not include any ABC system with pseudogenes present. ****>5 functional systems, ***3 or 4 functional systems, **2 functional systems, *1 functional system, — No functional systems.

**Table 4:** ABC system genes absent in at least one species when compared to *B. melitensis. *

Number	Family	Subfamily	Substrate/ Function	Type	*B. melitensis*	*B. abortus*	*B. suis*	*B. ovis*	*B. canis*
				IM	BMEI1851	−	+	+	+
5	CCM		Possibly heme export	IM	BMEI1852	−	+	+	+
				ABC	BMEI1853	−	+	+	+
6	CDI		Involved in cell division	IM	BMEI0073, ftsX	+	+	−	+
				ABC	BMEI0072, ftsE	+	+	−	+
7	CLS		O antigen export system	ABC	BMEI1416, rfbB	−	+	+	+
				IM	BMEI1415, rfbD	−	+	+	+
13	DPL	PRT	Proteases, lipase, S-layer protein export	OMP	BMEI1029	+	−	−	+
14	DPL	CHV	Beta-(1→2) glucan export	IM-ABC	BMEI0984	+	+	−	+
16	DPL	HMT	Involved in mitochondrial export systems	IM-ABC	BMEI1743	−	−	−	−
				IM-ABC	BMEI1742	−	−	+	−
22	FAE		Fatty acid export	IM-ABC	BMEII0976	+	−	+	+
				CYTP	BMEI1040	+	+	−	−
31	ISB (ABCX)		Iron/sulphur centre biogenesis	CYTP	BMEI1042	+	+	−	−
				ABC	BMEI1041	+	+	−	−
				ABC	BMEI0964	+	+	+	−
36	MKL		Involved in toluene tolerance	IM	BMEI0965, ttg2B	+	+	+	−
				SS	BMEI0963, ttg2C	+	+	+	−
				IM	BMEII0087	+	+	+	−
				IM	BMEI0361	−	−	−	−
60	o228		Unknown	MFP	BMEI0359	−	−	−	−
				ABC	BMEI0360	−	−	−	−
				IM	−	BruAb10085	+	+	−
61	o228		Unknown	MFP	−	−	−	−	BCAN_A1712
				ABC	−	BruAb10084	+	+	+
62	o228		Unknown	MFP	−	$	$	BOV_1617	−
				IM-ABC	−	$	$	+	BCAN_A0087

Excludes ABC systems involved in import; −: gene absent in the *Brucella* species; +: gene present in the *Brucella *species; $: pseudogene present in the *Brucella *species; Number: refers to ABC system number in the full inventories/alignments of *Brucella* ABC systems
